# Supplementary material for: Rhythmic light flicker rescues hippocampal low gamma and protects ischemic neurons by enhancing presynaptic plasticity
Source: Nat Commun. 2020 Jun 15;11:3012. doi: 10.1038/s41467-020-16826-0 (PMC7296037; doi:10.1038/s41467-020-16826-0)
Supplement: Supplementary file 1 — Supplementary Information [file 41467_2020_16826_MOESM1_ESM.pdf]

<u>Supplementary Information</u>

Rhythmic Light Flicker Rescues Hippocampal Low Gamma and Protects Ischemic Neurons by Enhancing Presynaptic Plasticity

Zheng et al.

SUPPLEMENTARY FIGURES

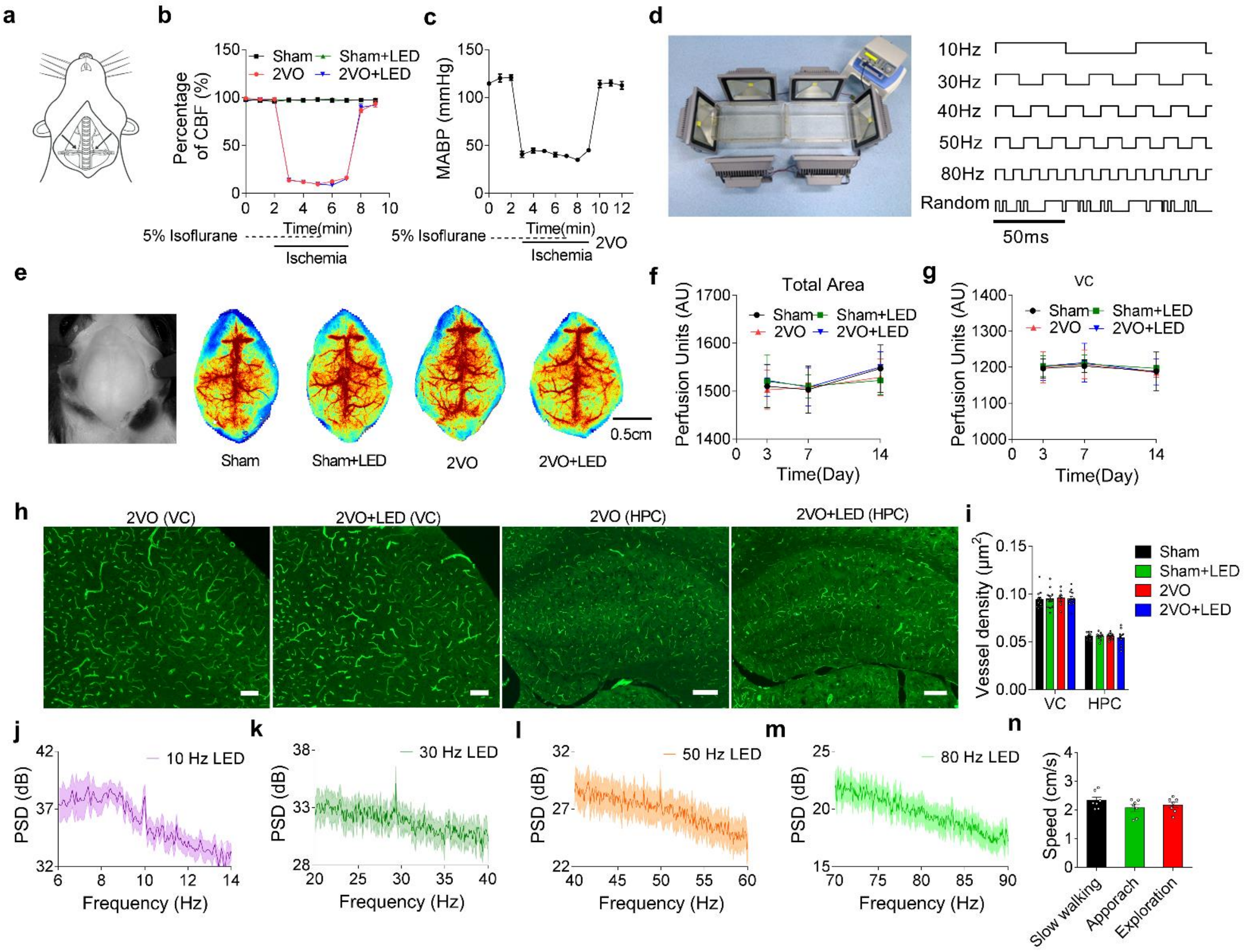

Supplementary Figure 1. 2VO model, blood flow, light flicker setup, and LFPs. **a** An illustration of mouse 2VO surgery site with two common carotid arteries indicated by the two arrows. **b** Reduction of hippocampal regional blood flow measured using a laser Doppler flowmeter (Sham, Sham+LED, 2VO+LED n = 3 mice each group respectively, 2VO n = 5 mice). **c** Systemic hypotension using 5% isoflurane measured by tail MABP (n = 5 mice). **d** A photograph showing the setup for light visual stimulation experiments (left panel) and traces of transistor-transistor logic pulses that turn the lights on (high) or off (low) (right panel). Example stimuli are shown, including 10 Hz, 30 Hz, 40 Hz, 50 Hz, 80 Hz, and random frequency flicker. For random stimulation, lights are on and off for a randomized interval > 0 that averages 12.5 ms. For example, random stimulation with off intervals that ranged from 0.2 to 34.1 ms was used. **e** Laser speckle flowmetry. The skin of the scalp was cut along the

sagittal plane to expose the braincase for imaging (first panel on the left). Examples of cerebral blood flow of four groups of mice. Scale bar = 0.5 cm. Laser speckle flowmetry quantification of total cerebral blood flow (**f**, *n* = 3 mice in each group) and visual cortex area (**g**, *n* = 3 mice in each group) of mice at 3 d, 7 d, and 14 d after 2VO or sham operations. **h** Images of coronal brain sections perfused with FITC-Lectin to show vascular density in areas of the hippocampus and visual cortex of 2VO and 2VO+LED mice 14 d after 2VO. Scale bars = 50 µm. **i** Quantification of vascular density based on lectin staining. No significant differences occurred amongst the four groups of mice (Sham: *n* = 12 slices, Sham +LED: *n* = 12 slices, 2VO: *n* = 8 slices, 2VO+LED: *n* = 12 slices in VC; Sham: *n* = 11 slices, Sham +LED: *n* = 10 slices, 2VO: *n* = 11 slices, 2VO+LED: *n* = 10 slices in HPC; the slices of each group was from 3 mice). **j** – **m** CA1 LFP power spectra of mice treated with 10 Hz, 30 Hz, 50 Hz, and 80 Hz light flicker. Noted a clear peak at corresponding theta and low gamma frequencies of 10 Hz, 30 Hz, and 50 Hz. There is a less clear response at the 80 Hz high gamma (**m**). **n** The speed/velocity of three locomotion states was measured at 2 cm/s ~ 3 cm/s forward (1ANOVA, $F_{(2, 18)} = 1.494$, $P = 0.2509$, *n* = 7 sessions / 3 mice each group). Data expressed as mean ± SEM. Error bars indicate SEM. The test used in **f, g**: RM-2ANOVA with Sidak's post hoc analysis; **i**: 2ANOVA with Tukey's multiple comparisons post hoc test. Source data are provided as a Source Data file.

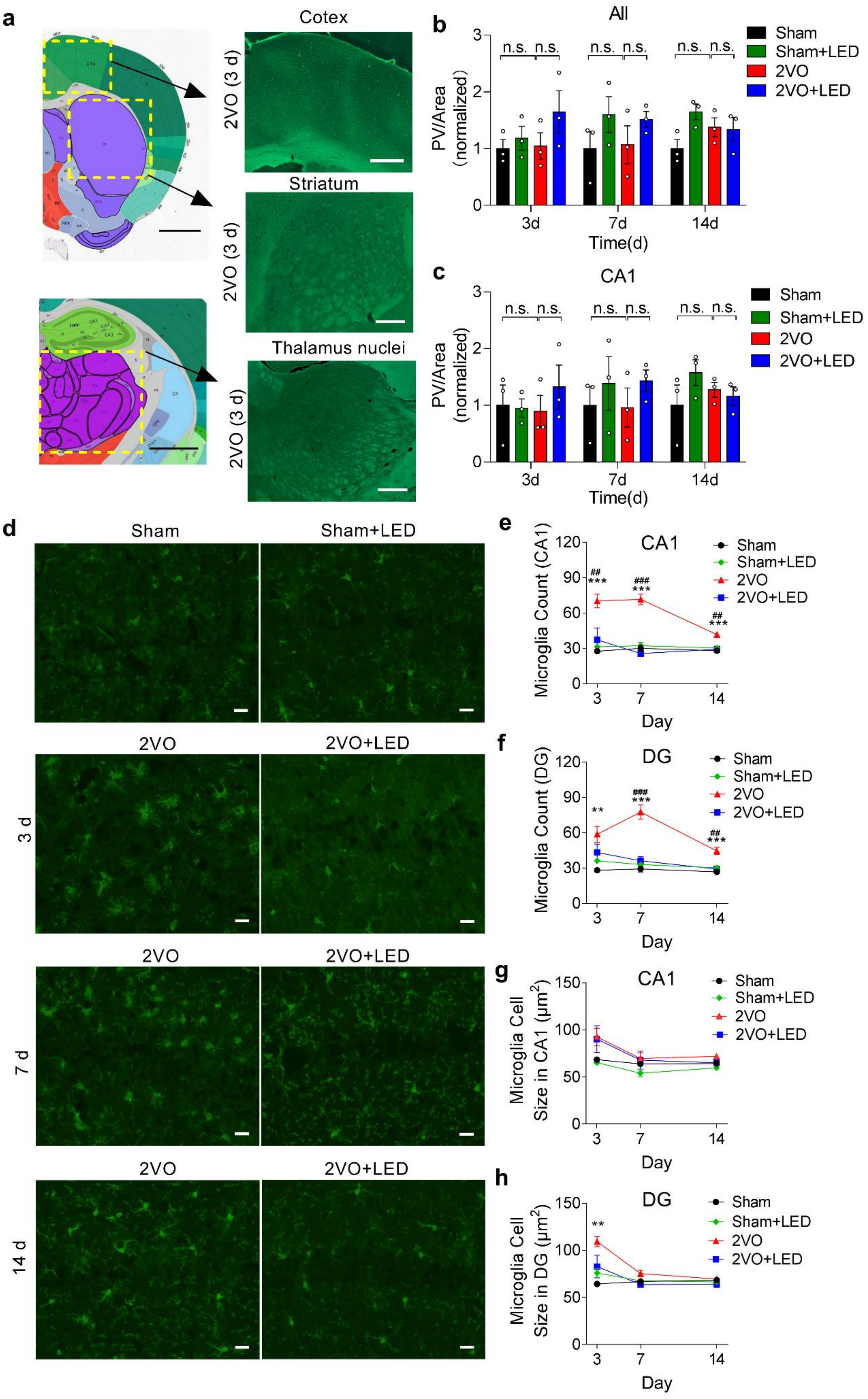
a
Cotex
Striatum
Thalamus nuclei
2VO (3 d)
b
All
c
CA1
n.s.
PV/Area (normalized)
3d
7d
14d
Time(d)
Sham
Sham+LED
2VO
2VO+LED
d
3 d
7 d
14 d
e
CA1
Microglia Count (CA1)
f
DG
Microglia Count (DG)
g
Microglia Cell Size in CA1 (μm2)
h
Microglia Cell Size in DG (μm2)
Day

Supplementary Figure 2. Characterization of 2VO injury and microglia response. a Mouse brain coronal sections at 3 d after 2VO were stained with FJB to show the absence of FBJ positive degenerating cells in the frontal cortex, striatum, and thalamus nuclei (Scale bars = 100 µm). Brain atlas was used as a reference to confirm the corresponding areas shown on the left (Scale bar = 1200 µm; http://atlas.brain-map.org/atlas?atlas=1&plate=100960240). **b** Quantification of parvalbumin-positive neurons in the hippocampus (**b**) and the CA1 region (**c**). **d** Representative images of coronal brain sections were subjected to IBA1 immunohistochemical staining to detect changes in microglia, and the statistical analyses were plotted in **e-h. e, f.** The 2VO surgery elicited a significant increase in the numbers of IBA1 positive microglia in the CA1 (2ANOVA, day 3, $F_{(1, 16)} = 9.605$, $P = 0.0069$; day7, $F_{(1, 16)} = 58.88$, $P < 0.0001$; day14, $F_{(1, 16)} = 14.67$, $P = 0.0015$, Tukey's post hoc test: Sham vs. 2VO, $P_{3day} = 0.0006$, $P_{7day} < 0.0001$, $P_{14day} = 0.0007$) and DG areas (2ANOVA, day 3, $F_{(1, 16)} = 5.302$, $P = 0.0351$; day7, $F_{(1, 16)} = 32.68$, $P < 0.0001$; day14, $F_{(1, 16)} = 16.41$, $P = 0.0009$, Tukey's post hoc test: Sham vs. 2VO, $P_{3day} = 0.003$, $P_{7day} < 0.0001$, $P_{14day} = 0.0004$) in 2VO group, but significantly reduced in 2VO+LED group (Tukey's post hoc test: 2VO vs. 2VO+LED: CA1: $P_{3day} = 0.0059$, $P_{7day} < 0.0001$, $P_{14day} = 0.0017$; DG: $P_{3day} = 0.1819$, $P_{7day} < 0.0001$, $P_{14day} = 0.0016$), which indicated an inverse correlation with neuroprotection **g, h** Quantification of the size of microglia from areas of CA1 (**g**) and DG (**h,** 2ANOVA, day 3, $F_{(1, 16)} = 7.405$, $P = 0.0151$, $P_{Sham\ vs.2VO} = 0.002$;). Scale bars = 50 µm. Data represent the mean ± SEM. Error bars indicate SEM. The asterisk sign (*) indicates a comparison between the sham and 2VO group, and the hash sign (#) indicates a comparison between 2VO and 2VO+LED group. n.s., not significant; **, ## $p < 0.01$; ***, ###$p < 0.001$. The test used in **b, c**, **e-h**: 2ANOVA with Tukey's post hoc test. $n = 3$ mice each group in **b** and **c** with each point representing the average of at least 7 slices per animal brain. $n = 5$ slices from 3 mice each group in **e-h**. Source data are provided as a Source Data file.

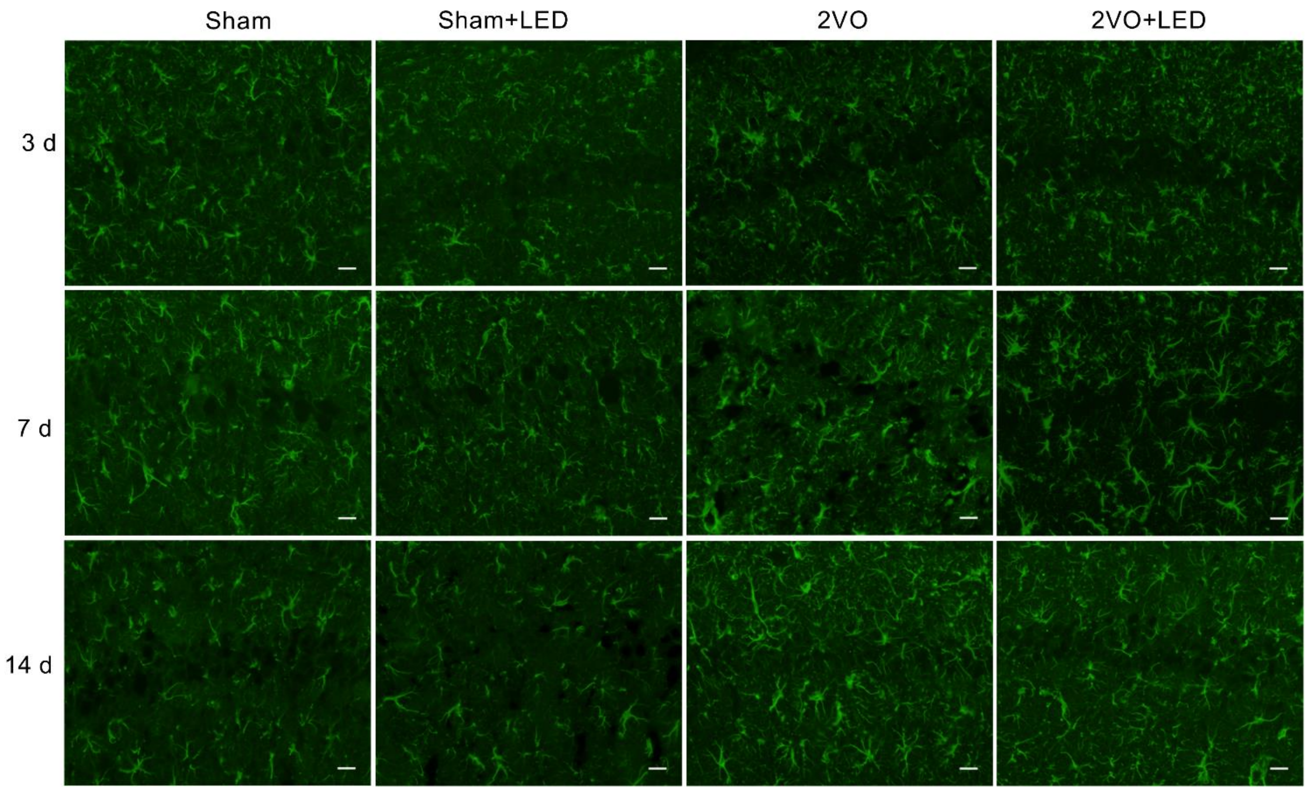

Supplementary Figure 3. Expression of GFAP in ischemic mouse hippocampus. Representative immunohistochemical staining for GFAP on coronal hippocampal brain sections (n = 3 mice per group). Scale bars = 50 µm.

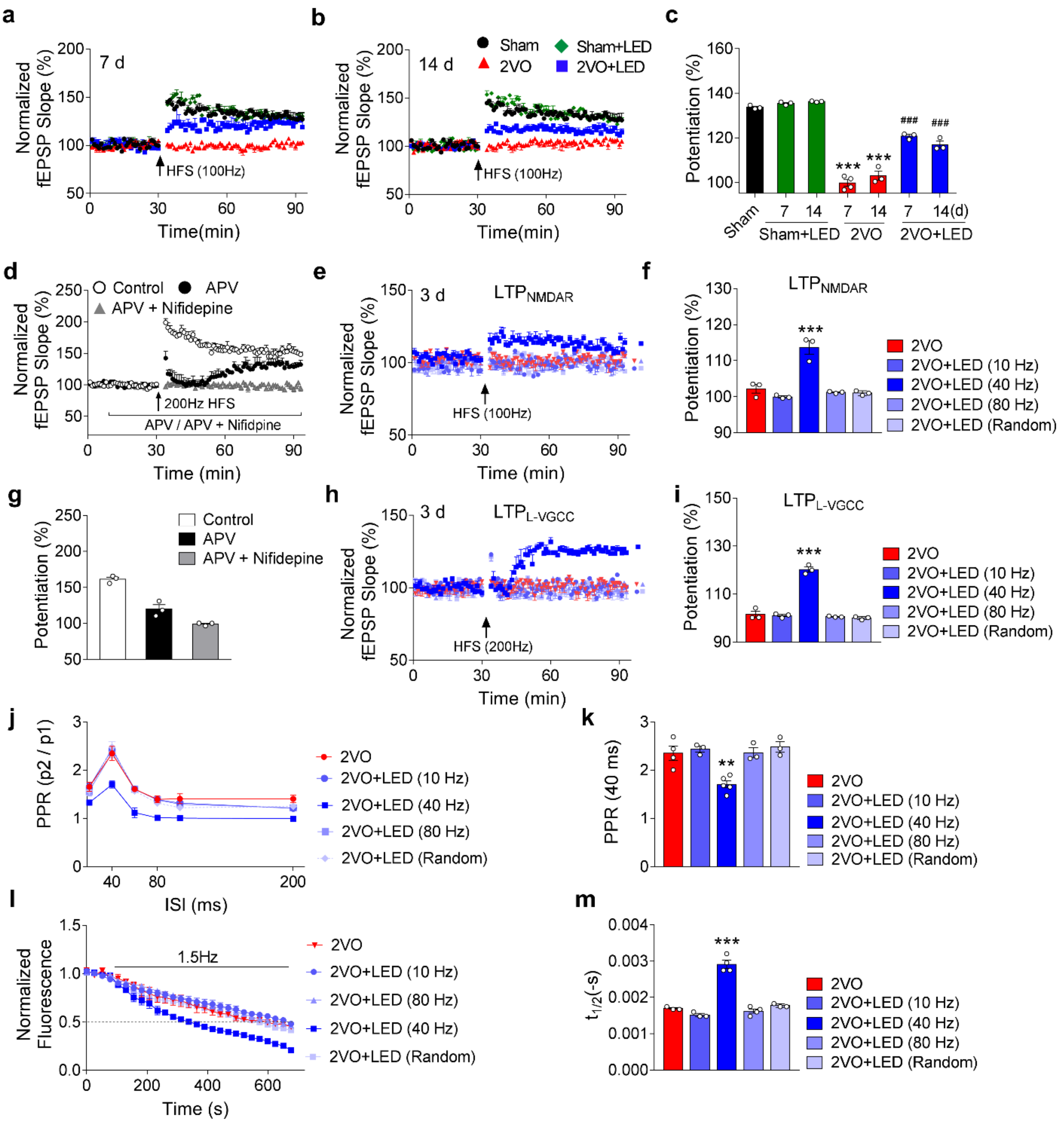

Supplementary Figure 4. Electrophysiology of LTPs and PPRs. Measurement of LTP_{NMDAR}, generated by 100 Hz frequency tetanic stimulation of the Schaffer collaterals projected to the CA1 pyramidal neurons on acute hippocampal slices of mice 7 d (**a**, RM-2ANOVA, Sham vs. 2VO: $F_{(1, 5)} = 362.2$, $P < 0.0001$; 2VO vs. 2VO+LED: $F_{(1, 5)} = 129$, $P < 0.0001$; n = 3 mice in each group except n = 4 mice in 2VO group), and 14 d (**b**, RM-2ANOVA, Sham vs. 2VO: $F_{(1, 4)} = 207.9$, $P = 0.0001$; 2VO vs. 2VO+LED: $F_{(1, 4)} = 28.71$, $P = 0.0059$; n = 3 mice each group) after 2VO surgeries. **c** The first 30 min of the evoked responses were used as the baseline responses to quantify LTP_{NMDAR} magnitude between 0 and 60 min after the HFS (2ANVOA, 7 d: $F_{(1, 9)} = 83.84$, $P < 0.0001$ and $P_{Sham\ vs.\ 2VO} < 0.0001$, $P_{2VO\ vs.\ 2VO+LED} < 0.0001$, n = 3 mice in each group except n = 4 mice in 2VO group; 14 d, $F_{(1,}$

$_{8)}$ = 18.37, P = 0.0027 and $P_{\text{Sham vs. 2VO}} < 0.0001$, $P_{\text{2VO vs. 2VO+LED}} = 0.0003$, n = 3 mice each group). **d** $\text{LTP}_{\text{L-VGCC}}$ induced with a 200 Hz tetanus (arrowhead) in the presence of 50 μM D-APV and 10 μM nifedipine compared with the control, and their quantification was shown in **g** (n = 3 mice each group). **e-f** Measurement of the magnitude of $\text{LTP}_{\text{NMDAR}}$ on hippocampal brain slices of 2VO and 2VO mouse subjected to light flicker at frequencies of 10 Hz, 40 Hz, 80 Hz, and a random frequency, respectively. Only 40 Hz light flicker was able to show significant $\text{LTP}_{\text{NMDAR}}$ (**e:** RM-2ANOVA, $F_{(1, 4)} = 25.26$, $P = 0.0074$; **f:** 1ANOVA with Dunnett's post hoc test $P_{\text{2VO vs. 2VO+LED(40 Hz)}} = 0.0001$; n = 3 mice each group). **h-i** $\text{LTP}_{\text{L-VGCC}}$ was induced by 40 Hz light flicker (blue-colored line in **h**), but not other frequencies of light flickers, as quantitated according to the responses between 0 and 60 min after the HFS (**h**, RM-2ANOVA, $F_{(1, 4)} = 120.1$, $P = 0.0004$; **i**, 1ANOVA with Dunnett's post hoc test $P_{\text{2VO vs. 2VO+LED(40 Hz)}} = 0.0001$; n = 3 mice in each group). **j** Hippocampal slices from 2VO and 2VO mice subjected to light flickers at the indicated frequencies were measured for PPR (**j**) and quantification. **k** The ratios of the slope of the second response to that of the first at the 40 ms ISI were quantified and plotted. The 40 Hz light flicker significantly reduced the PPR in 2VO mice (1ANOVA, $F_{(4, 13)} = 11.19$, $P = 0.0004$, Tukey's post hoc test $P_{\text{2VO vs 2VO+LED (40 Hz)}}$ = 0.0027. 2VO: n = 4, 2VO+LED (10 Hz): n = 3, 2VO+LED (40 Hz): n = 5, 2VO+LED (80 Hz): n = 3, 2VO+LED (Random): n = 3 mice). **l** Brain slices of 2VO and 2VO mouse exposed to the indicated frequencies of light flickers were subjected to measurements to determine the kinetics of $\text{FM}_{1\text{-}43}$ release from presynaptic terminals. **m** The rate of puncta unloading in brain slices ($1/t_{1/2}$) was plotted and shown, which suggested that 40 Hz light flicker has a significantly increased effect on vesicle release in 2VO mice (1ANOVA,$F_{(4, 12)}$ = 74.73, P < 0.0001, Tukey's post hoc test $P_{\text{2VO vs 2VO+LED (40 Hz)}} < 0.0001$. 2VO: n = 3, 2VO+LED (10 Hz): n = 3, 2VO+LED (40 Hz): n = 4, 2VO+LED (80 Hz): n = 4, 2VO+LED (Random): n = 3 mice). Data represent the mean ± SEM. Error bars indicate SEM. **p < 0.01; ***, ###p < 0.001. The test used in **a**, **b**, **e**, **h**: RM-2ANOVA with Sidak's post hoc test; **c:** 2ANOVA with Tukey's post hoc test; **f**, **i:** 1ANOVA with Dunnett's post hoc test; **k**, **m:** 1ANOVAwith Tukey's post hoc test. Source data are provided as a Source Data file.

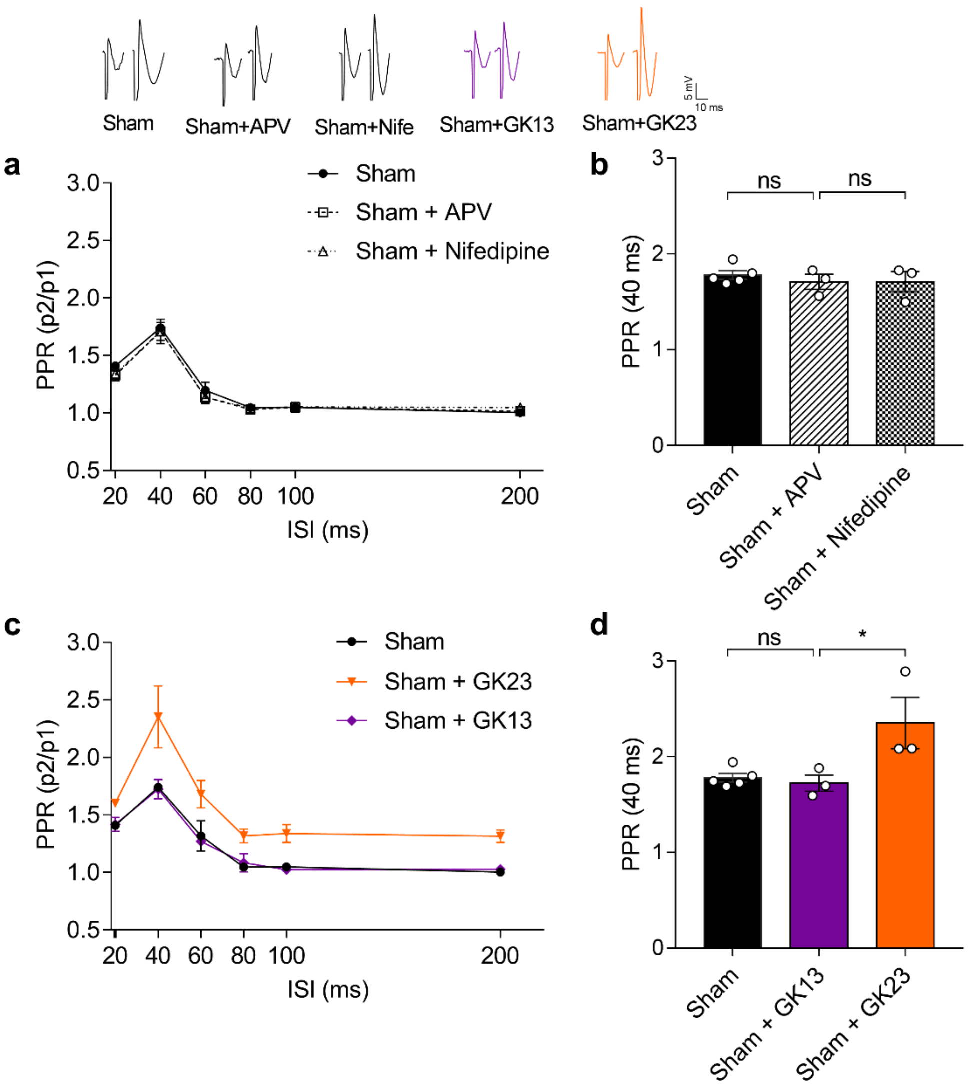

Supplementary Figure 5. Paired-pulse facilitation on hippocampal slices. Sham brain hippocampal slices were prepared for PPR recordings. PPR experiments were performed using inter-stimulus intervals (ISIs) of 20, 40, 60, 80, 100, and 200 ms. PPR (%) was estimated by the ratio of the second pulse-induced fEPSP slope to the first one (**a, b**). The raw tracings of the 1st and 2nd fEPSPs at 40 ms are shown on the top panel of **a**. PPR recordings in the presence of D-APV (50 µM) and Nifedipine (10 µM) (**a, b**, 1ANOVA, $F_{(2, 8)} = 0.3875$, $P = 0.6909$, Tukey's post hoc, $P_{\text{Sham vs. Sham+APV}} = 0.7538$, $P_{\text{Sham+APV vs. Sham+Nifedipine}} > 0.9999$. Sham: $n = 5$, Sham + APV: $n = 3$, Sham + Nifedipine: $n = 3$.), and PPR recordings on brain slices incubated with GK23 and GK13 1 h before the recordings (**c, d**, 1ANOVA, $F_{(2, 8)} = 6.011$, $P = 0.0255$, Tukey's post hoc, $P_{\text{Sham vs. Sham+GK23}} = 0.0358$, $P_{\text{Sham vs. Sham+GK13}} = 0.9488$. Sham: $n = 5$, Sham + GK13: $n = 3$, Sham + GK23: $n = 3$). Data expressed as mean ± SEM; Error bars indicate SEM. n.s., not significant; *, $p < 0.05$; Test used in **b, d**: 1ANOVA, Tukey's multiple comparisons post hoc test to identify significant group. Source data are provided as a Source Data file.

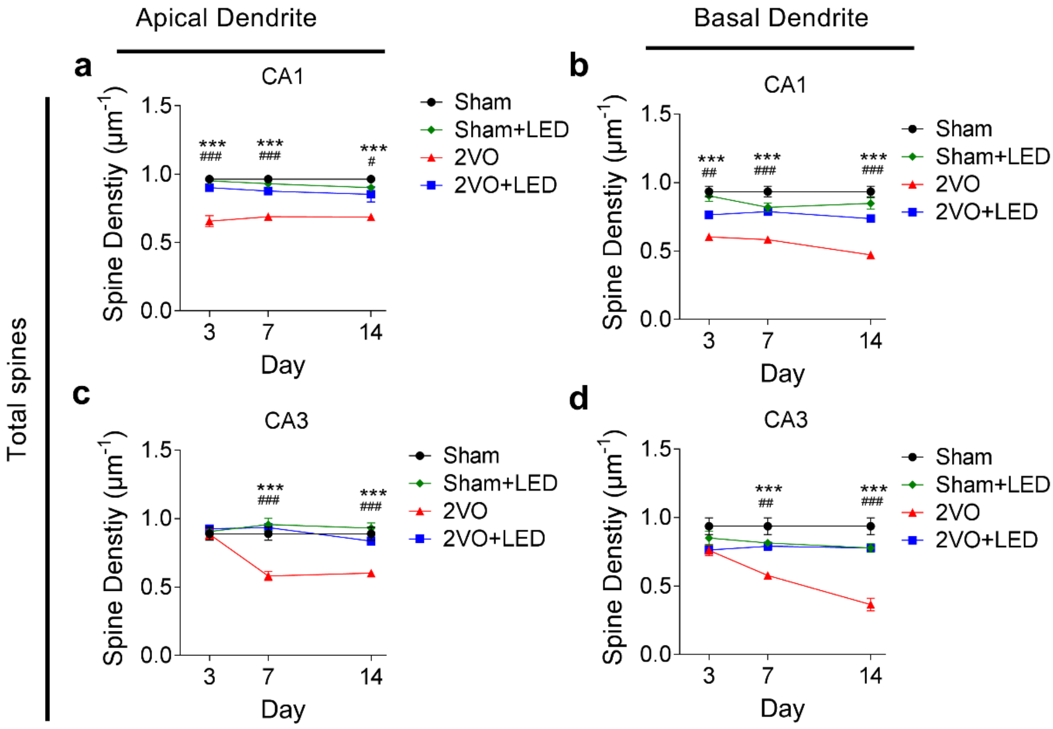

Supplementary Figure 6. Structural plasticity of CA3-CA1 pyramidal neurons. a-d, Quantifications of dendritic spines per unit length of the CA1 and CA3 apical and basal dendrites, respectively, at 3 d, 7d, and 14 d post-2VO surgery. **a** and **b** showed that 2VO caused a significant loss of CA1 apical and basal dendritic spines throughout the 14 d periods (Apical: 2ANOVA, day 3, $F_{(1, 20)} = 24.88$, $P < 0.0001$; day7, $F_{(1, 20)} = 32.49$, $P < 0.0001$; day14, $F_{(1, 20)} = 11.2$, $P = 0.0032$, Tukey's post hoc test: Sham vs. 2VO, $P_{3day} < 0.0001$, $P_{7day} < 0.0001$, $P_{14day} < 0.0001$; Basal: 2ANOVA, day 3, $F_{(1, 20)} = 9.045$, $P = 0.007$; day7, $F_{(1, 20)} = 28.18$, $P < 0.0001$; day14, $F_{(1, 20)} = 27.28$, $P < 0.0001$, Tukey's post hoc test: Sham vs. 2VO, $P_{3day} < 0.0001$, $P_{7day} < 0.0001$, $P_{14day} < 0.0001$), while 40 Hz light treatment completely prevented the loss of CA1 dendritic spines (2ANOVA with Tukey's post hoc test: 2VO vs.2VO+LED: Apical, $P_{3day} < 0.0001$, $P_{7day} < 0.0001$, $P_{14day} = 0.0129$; Basal: $P_{3day} = 0.01$, $P_{7day} = 0.0006$, $P_{14day} = 0.0001$); However, the loss of CA3 dendritic spines only appeared at 7 d and 14 d post 2VO (Apical: 2ANOVA, day7, $F_{(1, 20)} = 13.14$, $P = 0.0017$; day14, $F_{(1, 20)} = 7.001$, $P = 0.0155$, Tukey's post hoc test: Sham vs. 2VO, $P_{7day} = 0.0001$, $P_{14day} < 0.0001$; Basal: 2ANOVA, day7, $F_{(1, 20)} = 22.05$, $P = 0.0001$; day14, $F_{(1, 20)} = 43.09$, $P < 0.0001$, Tukey's post hoc test: Sham vs. 2VO, $P_{7day} < 0.0001$, $P_{14day} < 0.0001$), not in the 2VO+LED group (Tukey's post hoc test: Apical, 2VO vs.2VO+LED**:** $P_{7day} < 0.0001$, $P_{14day} = 0.001$, Basal : $P_{7day} = 0.0022$, $P_{14day} < 0.0001$, **c** and **d**). Data represent the mean ± SEM. Error bars indicate SEM. *n* = 6 slices / 3 mice per group. *indicates differences between the Sham and 2VO group; # indicates differences between the 2VO and 2VO + LED group. *n* = 3 in each group with at least 5 slices per animal brain. #*P* < 0.05, ##*P* < 0.01, ***,###*P* < 0.001. 2ANOVA with Tukey's post hoc analysis. Source data are provided as a Source Data file.

a

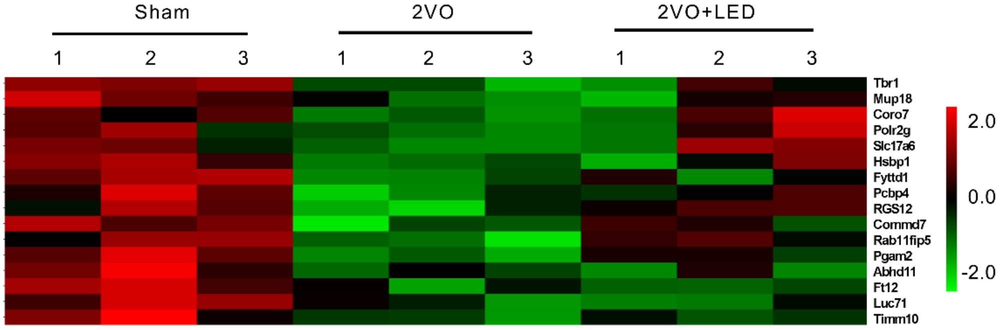

b

c

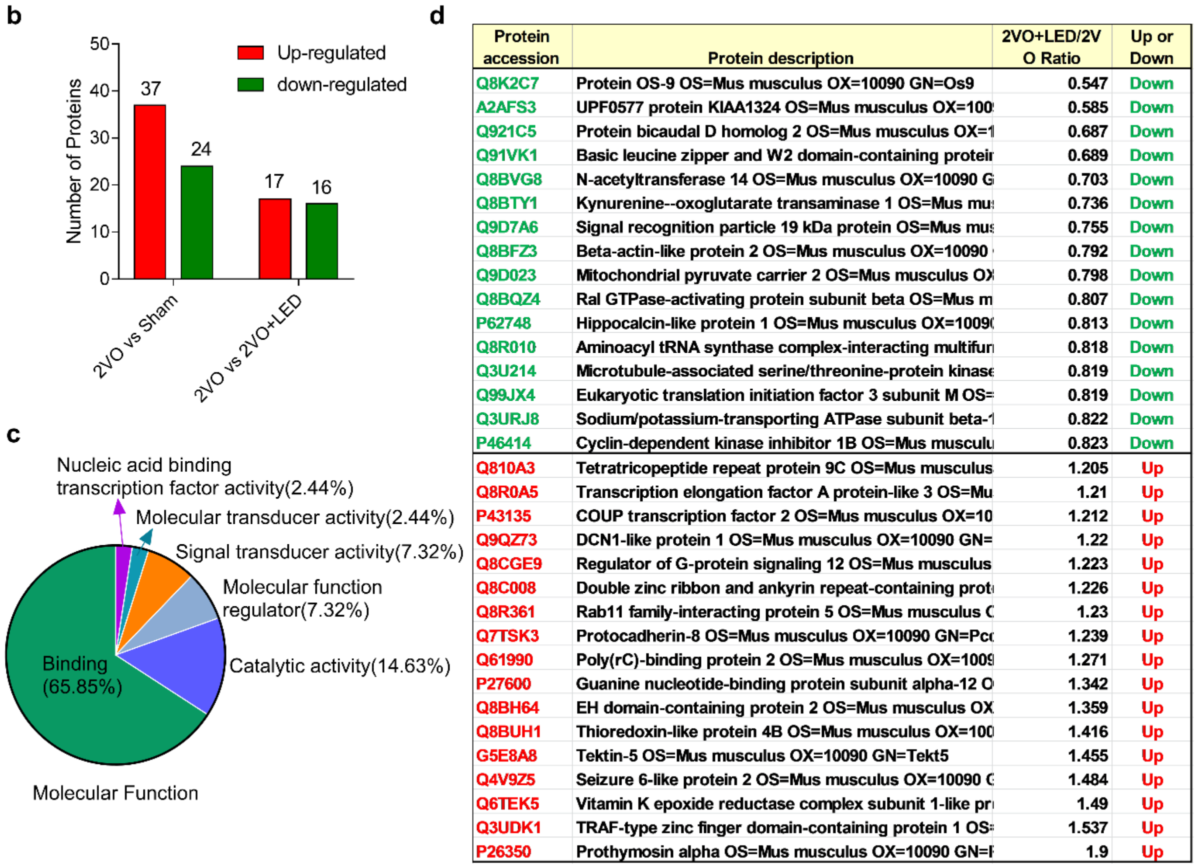

d

Protein accession	Protein description	2VO+LED/2VO Ratio	Up or Down
Q8K2C7	Protein OS-9 OS=Mus musculus OX=10090 GN=Os9	0.547	Down
A2AFS3	UPF0577 protein KIAA1324 OS=Mus musculus OX=100	0.585	Down
Q921C5	Protein bicaudal D homolog 2 OS=Mus musculus OX=1	0.687	Down
Q91VK1	Basic leucine zipper and W2 domain-containing protei	0.689	Down
Q8BVG8	N-acetyltransferase 14 OS=Mus musculus OX=10090 G	0.703	Down
Q8BTY1	Kynurenine--oxoglutarate transaminase 1 OS=Mus mu	0.736	Down
Q9D7A6	Signal recognition particle 19 kDa protein OS=Mus mu	0.755	Down
Q8BFZ3	Beta-actin-like protein 2 OS=Mus musculus OX=10090	0.792	Down
Q9D023	Mitochondrial pyruvate carrier 2 OS=Mus musculus O	0.798	Down
Q8BQZ4	Ral GTPase-activating protein subunit beta OS=Mus m	0.807	Down
P62748	Hippocalcin-like protein 1 OS=Mus musculus OX=1009	0.813	Down
Q8R010	Aminoacyl tRNA synthase complex-interacting multifu	0.818	Down
Q3U214	Microtubule-associated serine/threonine-protein kinas	0.819	Down
Q99JX4	Eukaryotic translation initiation factor 3 subunit M OS=	0.819	Down
Q3URJ8	Sodium/potassium-transporting ATPase subunit beta-1	0.822	Down
P46414	Cyclin-dependent kinase inhibitor 1B OS=Mus musculu	0.823	Down
Q810A3	Tetratricopeptide repeat protein 9C OS=Mus musculus	1.205	Up
Q8R0A5	Transcription elongation factor A protein-like 3 OS=Mu	1.21	Up
P43135	COUP transcription factor 2 OS=Mus musculus OX=10	1.212	Up
Q9QZ73	DCN1-like protein 1 OS=Mus musculus OX=10090 GN=	1.22	Up
Q8CGE9	Regulator of G-protein signaling 12 OS=Mus musculus	1.223	Up
Q8C008	Double zinc ribbon and ankyrin repeat-containing prot	1.226	Up
Q8R361	Rab11 family-interacting protein 5 OS=Mus musculus C	1.23	Up
Q7TSK3	Protocadherin-8 OS=Mus musculus OX=10090 GN=Pc	1.239	Up
Q61990	Poly(rC)-binding protein 2 OS=Mus musculus OX=1009	1.271	Up
P27600	Guanine nucleotide-binding protein subunit alpha-12 O	1.342	Up
Q8BH64	EH domain-containing protein 2 OS=Mus musculus OX	1.359	Up
Q8BUH1	Thioredoxin-like protein 4B OS=Mus musculus OX=100	1.416	Up
G5E8A8	Tektin-5 OS=Mus musculus OX=10090 GN=Tekt5	1.455	Up
Q4V9Z5	Seizure 6-like protein 2 OS=Mus musculus OX=10090 G	1.484	Up
Q6TEK5	Vitamin K epoxide reductase complex subunit 1-like pr	1.49	Up
Q3UDK1	TRAF-type zinc finger domain-containing protein 1 OS	1.537	Up
P26350	Prothymosin alpha OS=Mus musculus OX=10090 GN=	1.9	Up

Supplementary Figure 7. Proteomic analysis of ischemic mouse brain. a An excerpt from the heatmap showing a supervised clustering analysis of proteins identified in the hippocampus of 3 d post 2VO and 2VO+LED groups (n = 3 per group as indicated on the top). The red color indicated increased protein expression, and the green color indicated decreased protein expression. Clustering was performed on $\log_2$ transformed spectral counts from 3 replicated analyses (n = 3 mice). **b** A bar graph showing altered expression of all proteins changed more than ± 1.2-fold between indicated groups. **c** The GO analysis of these proteins based on the molecular functions of their expressions. **d** A list of proteins up-or down-regulated compared between the 2VO and 2VO+LED group of mice.

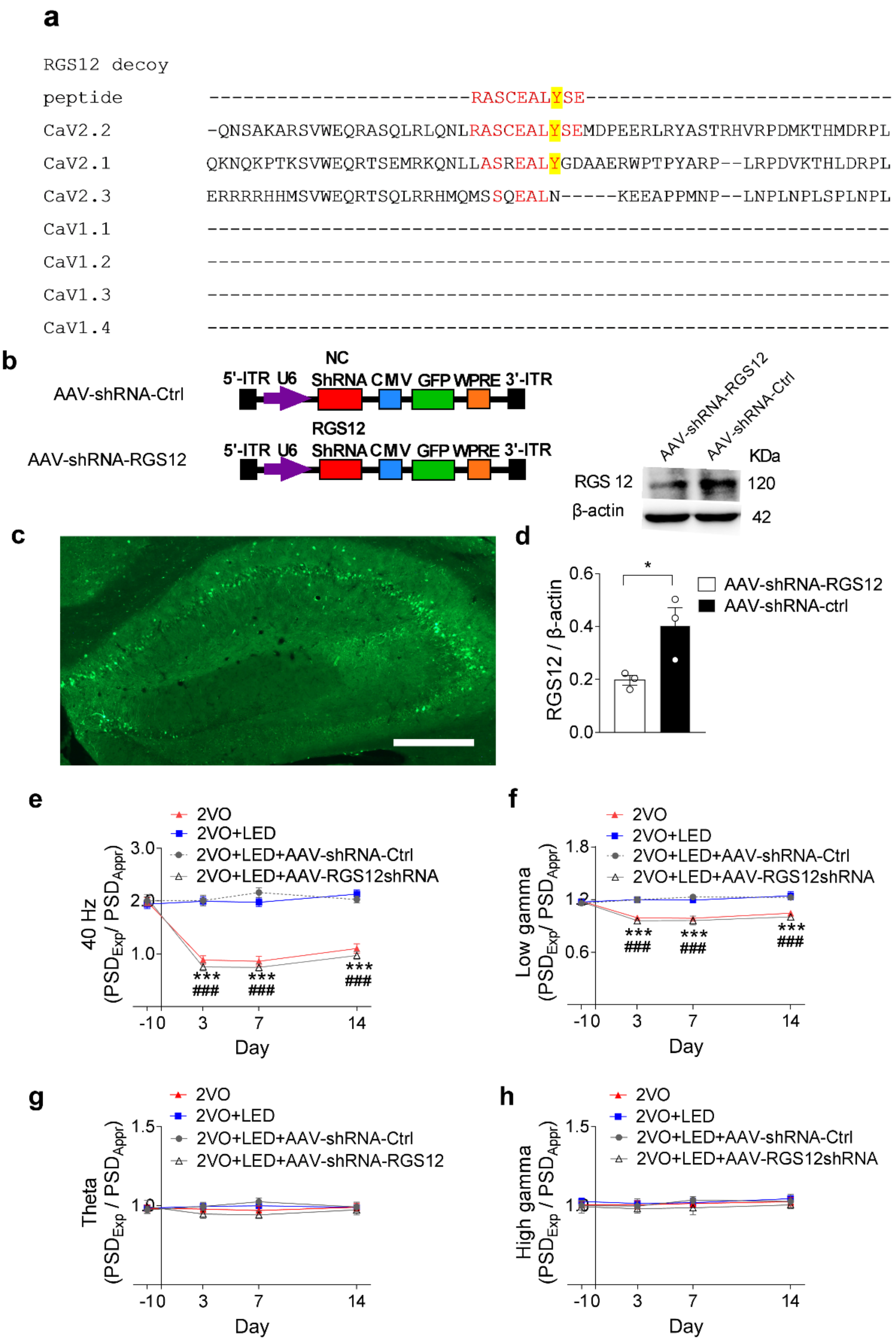

Supplementary Figure 8. Knockdown RGS12 expression. a Protein sequence alignment using CLUSTALW Multiple Sequence Alignment (https://www.genome.jp/tools-bin/clustalw) amongst RGS12 decoy peptide, Cav1.1, Cav1.2, Cav1.3, Cav2.2, and Cav2.3. RGS12 decoy

peptide sequence 100% aligned with that from $Ca_V2.2$ (N-VGCC). In contrast, the L-VGCC family members ($Ca_V1.1$, $Ca_V1.2$, $Ca_V1.3$, and $Ca_V1.4$) have no sequence homology to the RGS12 decoy peptide sequence. **b** Structures of recombinant adeno-associated viral vectors (AAV). **c** Representative images of the pyramidal neurons expressing AAV-shRNA-RGS12 (green) in the CA1 area (4 repeats from *n* = 4 mice in 2VO+LED+AAV-shRNA-RGS12 group). Scale bar = 100 μm. **d** The reduced expression of RGS12 in the hippocampus (approximately 50%) after expressing the AAV-shRNA-RGS12 vector for 3 weeks (*n* = 3 mice each group; The blot showing three independent repeats with similar results). Mice expressing the AAV-shRNA-RGS12 vector after 3 weeks were subjected to CA1 LFP *in vivo* recordings when approached and explored a novel object. **e-h** The ratio of PSD between Exploration (PSD_{Exp}) and Approach (PSD_{Appr}) of the four groups of mice at the indicated oscillation frequencies on the y-axis (**e**, 2VO+LED vs. 2VO+LED+AAV-shRNARGS12: $F_{(1, 8)}$ = 130.3, $P < 0.0001$ and $P_{3d} < 0.0001$, $P_{7d} < 0.0001$, $P_{14d} < 0.0001$; 2VO+LED+AAV-shRNA-Ctrl vs. 2VO+LED+AAV-shRNARGS12: $F_{(1, 6)}$ = 189.9, $P <$ 0.0001 and $P_{3d} < 0.0001$, $P_{7d} < 0.0001$, $P_{14d} < 0.0001$. **f**, 2VO+LED vs. 2VO+LED+ AAV-shRNARGS12: $F_{(1, 8)} = 42.71$, $P = 0.0002$ and $P_{3d} < 0.0001$, $P_{7d} < 0.0001$, $P_{14d} < 0.0001$; 2VO+LED +AAV-shRNA-Ctrl vs. 2VO+LED+AAV-shRNARGS12: $F_{(1, 6)} = 28.73$, P = 0.0017 and $P_{3d} = 0.0004$, $P_{7d} < 0.0001$, $P_{14d} = 0.0009$; **g**, 2VO+LED vs. 2VO+LED+AAV-shRNARGS12: $F_{(1, 8)} = 2.282$, $P = 0.1693$; 2VO+LED+AAV-shRNA-Ctrl vs. 2VO+LED+AAV-shRNARGS12: $F_{(1, 6)} = 2.324$, $P = 0.1783$. **h**, 2VO+LED vs. 2VO+LED+AAV-shRNARGS12: $F_{(1, 8)} = 1.949$, $P = 0.2002$; 2VO+LED+AAV-shRNA-Ctrl vs. 2VO+LED+ AAV-shRNARGS12: $F_{(1, 6)} = 0.8496$, $P = 0.3922$. 2VO: *n* = 6 sessions/ 3mice, 2VO+LED: *n* = 6 sessions/ 3mice, 2VO+LED+AAV-shRNA-Ctrl: *n* = 4 sessions/ 4mice, 2VO+LED+AAV-shRNA-RGS12: *n* = 4 sessions/ 4mice). Data represent the mean ± SEM. Error bars indicate SEM. ***, ###$P < 0.001$. The test used in **e-h**: RM-2ANOVA with Sidak's post hoc analysis. Source data are provided as a Source Data file.
